# The prevalence and risk factors for mental distress among Syrian refugees in Germany: a register-based follow-up study

**DOI:** 10.1186/s12888-020-02746-2

**Published:** 2020-07-08

**Authors:** Andrea Borho, Andre Viazminsky, Eva Morawa, Gregor Martin Schmitt, Ekaterini Georgiadou, Yesim Erim

**Affiliations:** 1 Department of Psychosomatic Medicine and Psychotherapy, Friedrich-Alexander University Erlangen-Nürnberg (FAU), Erlangen, Germany; 2 Erlangen City Council, Jobcenter, Erlangen, Germany; 3 Department of Psychiatry and Psychotherapy, Paracelsus Medical University, Nuremberg, Germany

**Keywords:** Syrian refugees, Germany, Follow-up, Register-based, Post-traumatic stress disorder, Depression, Generalized anxiety disorder, Mental distress, Mental health

## Abstract

**Background:**

Mental disorders among refugees as well as their risk factors are already well documented in cross-sectional reports. However, longitudinal follow-up designs are widely lacking. Therefore, the aim of this study was to examine the change of the prevalence of mental disorders among Syrian refugees with German residence permission, taking into account their increasing length of stay in Germany, and to uncover the change in their relationship to pre- and post-migration risk factors.

**Methods:**

This study formed part of a register-based follow-up study with two measurement points in Erlangen (Germany). At the first time of recruitment in 2017, 200 of the 518 Syrian refugees with residence permission living in Erlangen took part. During the second survey timeframe 1.5 years later, in 2019, 108 of the former 200 Syrian refugees participated again and formed the total sample for this follow-up study. The survey instruments included demographics, migration-related variables and symptoms of post-traumatic stress (Essen Trauma Inventory, ETI), depression (Patient Health Questionnaire - PHQ-9) and generalized anxiety disorder (GAD-7).

**Results:**

At the time of the first survey, 26.9% of the participants exceeded the cut-off for a clinically relevant depression diagnosis, 16.7% for an anxiety disorder and 13.9% for a PTSD diagnosis. At the second measurement point, it was 30.6% for depression, 15.7% for an anxiety disorder and 13.0% for PTSD. No significant changes between the measurement points were found for any of the disorders. In multiple linear regression analyses, higher perceived discrimination, a higher number of traumatic experiences and a shorter duration of residence permission were shown to be the most important pre- and post-migration predictors of psychological stress independent of the time of measurement.

**Conclusions:**

There is strong empirical evidence that the prevalence rates of mental distress among refugees are significantly higher compared to the overall population. However, it has not yet become clear how these prevalence rates change with an increasing length of stay in the host countries. The results of our study indicate that the psychological burden on this refugee population remains consistently high over time, despite partly improved living conditions, and confirm the importance of therapeutic interventions.

## Background

The civil war that has afflicted Syria since 2011 has forced 6.7 million people to leave their home country, making this one of the largest refugee displacements in recent history [1]. Most of them have fled to the neighbouring countries of Turkey (3.7 million), Lebanon (924.000) and Jordan (657.000). However, more than one million Syrian war refugees were seeking protection in Europe - of them, about 770.000 in Germany [2].

Upon arriving in their receiving countries, Syrian refugees may have experienced several war-related stressors such as imprisonment, torture, the death of loved ones as well as the destruction of their homes and livelihoods [3, 4]. In addition, they may have undertaken a dangerous and traumatizing escape to leave their homes and families in the hope of a better life in an unknown country [5]. In a cross-sectional study among 352 Syrian refugees in Turkey the average number of experienced traumatic events was 3.7 (SD = 1.9). In this study sample, the three most frequently mentioned traumatic experiences were “had been in a region that is affected by war” (92%), “experienced/witnessed the death of a close friend or a family member” (66%) and “saw and touched dead bodies apart from funerals” (51%) [6]. Another study conducted in Sweden with 1.215 Syrians revealed that 85% had experienced war at close quarters and 79% had been exposed to other life-threatening situations during their lives [7].

Besides these traumatic experiences, refugees are also confronted with post-migration stressors in their host countries, such as loss of their social lives, discrimination, poor integration, lack of relevant language and cultural skills, barriers to employment and uncertainty about their asylum status [4, 7–10]. In a study conducted in the U.S. among Syrian refugees the top three post-migration challenges were identified as being the language barrier, worries about family members back home and inability to pay living expenses [11].

Due to these traumatizing and stressful factors, Syrian refugees are at high risk of emotional disorders, in particular post-traumatic stress disorder (PTSD), depression and anxiety disorders [6, 12, 13]. In addition to the incidence of one of these disorders alone, studies have revealed a frequent co-occurrence of these diseases in refugee populations [9, 14, 15]. Studies tackling populations of Syrian refugees in different receiving countries vary and often end up with heterogeneous results in prevalence rates of depression (34.7–65%) [11, 16–22], PTSD (19.6–84%) [4, 6, 11, 16, 19, 20, 22, 23], and unspecified anxiety disorder (36.1–60%) [11, 16, 19, 22]. This can mainly be explained due to clinical and methodological factors, e.g. the use of different assessment instruments to record mental distress, or varying diagnostic criteria and cut-off points. Other reasons for the diverging results may be the inconsistent living conditions in the receiving countries, the different measurement times after arrival in the host country, or the specific sample characteristics. These factors lead to a difficult comparison of the study outcomes. However, according to several study results and meta-analyses pooling data from research with refugees originating from multiple countries resettling in western and non-western nations, refugees suffer up to ten times more frequently from anxiety disorders, depression or PTSD than the general population [14, 15, 24, 25].

Regarding previous studies investigating risk factors of mental disorders in Syrian refugees, a predominant focus on the impact of experienced traumatic events stands out. Research evidence suggests that there is a strong positive correlation between the number of traumatic events and emotional disorders, especially post-traumatic stress symptoms [4, 20, 26, 27]. Alpak et al. [6] for instance found that Syrian refugees who experienced two or more traumatic events were at higher risk of developing PTSD than others. In another study among 781 Syrian refugees in a refugee camp in Turkey predictors for PTSD were being female, having experienced life threatening events, and injury of a loved one. Predictors for elevated depressive symptoms were being female, having a loved one who was tortured, and not being satisfied with the current living situation [20]. These results indicate that besides the traumatic experiences at home or whilst fleeing their country, resettlement in a new country may also put refugees under great strain and have a powerful impact on mental health [19]. In this regard, studies have indicated that post-migration experiences often tend to be more detrimental to refugees’ mental health than pre-migration traumatic events and that the post-migration environment plays a key role in either fostering or impeding recovery from trauma [28]. Furthermore, Schick et al. showed that psychological impairment in traumatized refugees is associated with poor integration. The authors suppose, refugees suffering from trauma may have difficulties in regulating the ongoing effects of stress on their functional capacity, and the effects of post-migration difficulties contribute to ongoing mental health problems [29]. Regarding specific post-migration stress factors, in a study with asylum seekers in Australia, anxiety scores were associated with post-migratory poverty, while PTSD was correlated with delays in processing refugee applications, difficulties in dealing with immigration officials, obstacles to employment, and racial discrimination [9]. To date, the effects of post-migration stressors, such as living environment, racial discrimination or duration of further residence permission on mental disorders of the Syrian refugee population have not been sufficiently examined.

Another factor that has hardly been studied so far is the influence of refugee’s duration of stay in the host country on mental health. A previous study by our group on Arabic-speaking asylum seekers (34% Syrians) residing in collective accommodation centers showed that participants with mental distress had shorter periods of stay in Germany compared to the asylum seekers without mental distress [3]. A systematic review of 29 studies on the long-term mental health of adult refugees worldwide, however described the persistent high risk of mental health problems, even after five or more years following displacement, with emotional distress prevalence estimates in the range of 20% and above [30]. Heterogeneous results have been found in a German 6-month follow-up study among 34 refugees from different countries of origin, living in an admission center. Between the first and second measurement point, the rate of psychiatric diagnoses decreased from 74 to 38%, including depression and anxiety. In contrast, the number of PTSD cases increased [31]. The study results just described show that with regard to the impact of length of stay in the resettlement country on mental health, the state of research is still quite inconsistent.

In previous studies, the prevalence of mental disorders as well as the role of pre- and post-migration stressors on refugee’s mental health have mainly been documented in cross-sectional reports. To date, longitudinal follow-up designs are widely lacking. To the best of our knowledge, the presented register-based follow-up study is the first to examine the prevalence and risk factors of mental distress among Syrian refugees with residence permission and increasing length of stay in Germany. This study aims to gain an insight into possible evolutions in Syrian refugees’ mental health, living conditions and stress factors. We therefore focus on Syrian refugees who fled to Germany between 2015 and 2017 and survey depression, anxiety and post-traumatic stress with an interval of about 1.5 years. In the first part of this survey, conducted in 2017, symptoms of PTSD were found in 11.4% of the 200 participating Syrian refugees with residence permission. Clinically relevant depressive symptoms were confirmed in 27% and generalized anxiety in 13.5% of the sample. The criteria for at least one diagnosis were met by 30.5% of the participants. More severe PTSD symptoms were associated with older age, shorter validity of the residence permission and larger number of traumatic events. Depression symptoms were associated with younger age, shorter duration of escape journey and larger number of traumatic events. Generalized anxiety symptoms correlated with female gender. The detailed results have been published in Georgiadou et al. [32]. Regarding these baseline results, we would now like to take the next step to uncover possible changes in these prevalence rates and stress factors. More precisely, the aims of our study were to examine the prevalence of mental disorders, post-migratory stress factors and living conditions of Syrian refugees with residence permission and increasing length of stay in Germany over a period of 1.5 years. In addition, we aimed to find out which of the identified pre- and post-migration risk factors influence Syrian refugees’ mental distress.

## Methods

This investigation was part of a prospective register-based study with two measurement times on mental health and integration of Syrian refugees (PROSREF) in Erlangen, Germany. It was designed together with the local authorities of the city Erlangen, in particular with the job center.

**Fig. 1:**
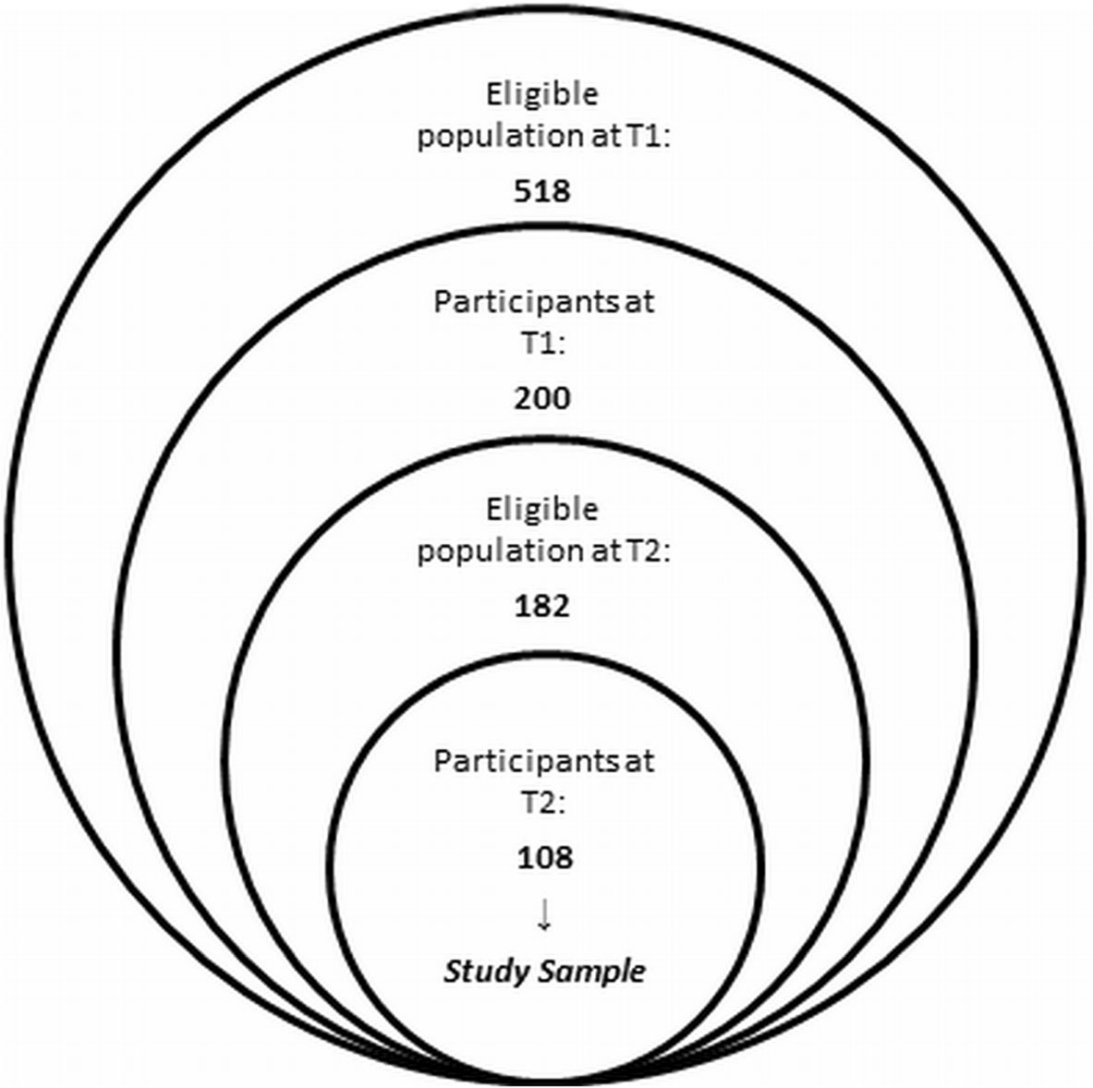
Study sample selection. *Note.* T1 = measurement time 1 in 2017, T2 = measurement time 2 in 2019

For the presented analysis we conducted a 1.5 year two-wave longitudinal prospective register-based study and followed Syrian refugees with residence permission in Germany. At the time of the first measurement point (T1) in 2017, we recruited 200 of the 518 (38.61%) Syrian refugees registered at the job center Erlangen. All participants were resident in the city of Erlangen, receiving unemployment benefits and in possession of residence permission. Study inclusion criteria for T1 were: age above 18, arrival in Germany after 2014, registration at the job center Erlangen, agreement to participate in the study and a good knowledge of the Arabic language (at least spoken). This population was chosen because of their homogeneity in language and culture. For the invitation to participate in the study, the job center Erlangen informed all 518 registered Syrian refugees about this study by post. At the second measurement point (T2), about one and a half years later in 2019, only the 182 Syrian refugees were contacted who had already participated in T1 and were still registered at the job center Erlangen. Of these, 108 participants attended (59.34%) and represented the total sample of our study. The process of study sample selection is presented in Fig. 1.

At both measurement times, all participants were invited to attend pre-arranged meetings in a room at the Erlangen city hall. Each participant was offered several dates on which he/she could participate in the study. After being informed about the study course and goals, participants gave their consent and completed the questionnaires described in the section “assessment instruments”. We obtained informed consent according to the general principles by providing native language translated information and a consent form. At least one German (psychologist) and one Arabic (medical doctoral student) speaking team member remained in the room and offered further explanations if needed. Thirteen of the 108 participants (12.0%) needed help from the Arabic-speaking team member to complete the questionnaires. In the room, a maximum of 16 participants completed the questionnaires at the same time.

T1 was conducted between July and December 2017 and T2 between February and April 2019. Participation in the study was voluntary and all of the study participants received a reimbursement of 15€ at T1 and of 20€ at T2. The study was approved by the Ethics Committee of the Medical Faculty of the Friedrich-Alexander University Erlangen-Nürnberg (FAU) (project identification code: 74_17 B).

### Assessment instruments

During both measurement points, the survey instruments included the same sociodemographic and migration-related variables, as well as perceived discrimination, depressive symptoms, symptoms of generalized anxiety disorder and symptoms of post-traumatic stress disorder. Additionally at T2, participants rated the change of their general health status and their mental health status from T1 to T2 on a five-point Likert scale from “much worse than last time” (0) to “much better than last time” (4). The questions on sociodemographic and migration-related variables were translated by two independent translators from German into Arabic, back-translated and finally combined into a final version [32]. Comprehensibility and cultural validity of the assessment instruments were previously tested in Georgiadou et al. (2017) [3]. Information on the current employment status was obtained from the cooperating job center.

#### Discrimination

Using a self-developed questionnaire, used in a previous study by our team [33], perceived discrimination in four domains of daily life, neighborhood, shops, authorities and professional life were rated on a scale from “never” (0) to “always” (4). The level of generally perceived discrimination was calculated on the basis of the mean levels of all four categories, with higher values representing a higher perception of discrimination. Cronbach’s Alpha was α = 0.845 for T1 and α = 0.866 for T2.

#### Depression (Patient Health Questionnaire – depression module; PHQ-9)

Depressive symptoms were measured by the Arabic version of the PHQ-9 [34, 35], which incorporates each of the nine DSM-IV [36] depression diagnostic criteria. The severity of depressive symptoms within the past 2 weeks is rated on a scale from “not present at all” (0) to “present nearly every day” (3). The possible range of the resulting sum score is 0 to 27 points. Sum scores of 10, 15 and 20 represent cut-offs for moderate, moderate to severe and severe levels of depressive symptoms. The clinically relevant cut-off for depressive symptoms is 10. Cronbach’s α in the present study sample was α = 0.892 for T1 and α = 0.897 for T2.

#### Generalized anxiety disorder (generalized anxiety disorder scale; GAD-7)

The Generalized Anxiety Disorder Scale is a seven item self-reported questionnaire considering the severity of various symptoms of generalized anxiety disorder (GAD) [37, 38]. Severity is rated on a 4-point Likert scale ranging from “not at all” (0) to “present nearly every day” (3) during the past 2 weeks. Sum scores can be assigned to severity of GAD symptoms using the following scheme: ≥ 5 mild, ≥ 10 moderate (cut-off for clinical relevance), and ≥ 15 severe anxiety symptom levels. In this study the Arabic version of this questionnaire was employed, which obtained a Cronbach’s Alpha of 0.907 for T1 and 0.918 for T2.

#### Post-traumatic stress disorder (Essen Trauma Inventory; ETI)

The Arabic version of the Essen Trauma Inventory [39] is a self-rating questionnaire that contains a list of potentially traumatic experiences and items to assess post-traumatic stress disorder according to criteria provided in DSM-IV (Diagnostic and Statistical Manual of Mental Disorders) [36]. By fulfillment of a traumatic experience, questions about objective and subjective threat of life (criterion A1 and A2) and 17 questions on PTSD symptoms are asked. These symptoms are rated on a 4-point Likert scale ranging from “never” (0) to “very often” (3). Clinically apparent PTSD is indicated by the experience of at least one traumatic event, the fulfillment of both criteria (A1 and A2) and a PTSD symptom list sum score (intrusion, avoidance and hyperarousal) of ≥ 27. In this study, internal consistency was α = 0.961 for T1 and α = 0.949 for T2.

### Statistical analysis

Statistical analysis was performed using IBM SPSS Statistics 21 (IBM Corporation, Armonk, New York). After analyzing missing values with Little’s MCAR test [40], values that were missing completely at random were replaced using expectation maximization method [41]. To profile the sociodemographic and migration-specific characteristics of the sample, the following descriptive statistics were computed for both measurement times: means, standard deviations, ranges and frequencies. Prevalence rates were calculated on the basis of available cut-off scores for each questionnaire. Comparisons between the two measurement times were analyzed using paired sample t-tests for continuous variables, the Wilcoxon signed-rank test for ordinal variables (and if values were not normally distributed) and the McNemar’s test for dichotomous variables. To avoid multicollinearity in regression analyses, bivariate correlations were computed among the main psychological variables. There were no correlation coefficients higher than 0.70 so that none of the tested variables had to be excluded from the multiple regression models. After testing for the assumptions, multiple linear regression analyses with the enter method were used to explore the influence of sociodemographic and migration-related variables on the severity of mental stress at T1 and T2 separately. Significance level in all analyses was predetermined at *p* ≤ 0.05.

## Results

### Non-responder analyses

Non-responder analyses for T1 are presented in Georgiadou et al. (2018) [32].

At T2 92 of the originally 200 Syrian refugees at T1 did not participate any more. The non-responders of T2 (M = 30.47, SD = 9.44) were significantly younger than those who participated at both measurement times (M = 35.67, SD = 10.93; t_(198)_ = 3.57, *p* < 0.001). There were no significant differences in gender (men non-responder T2: 68.5%; men responder T2: 66.3%; *C* = 0.023, *p* = 0.744; Mc Nemar’s test) or years of education (responder T2: M = 9.97, SD = 4.63; non-responder T2: M = 10.51, SD = 4.33; t_(184)_ = − 0.82, *p* = 0.418). There was no significant difference in the average score for depression of non-responders of T2 and responders. Measured at T1, the average score of depression for those who responded at both measurement times was M = 8.29 (SD = 6.34) and of non-responders of T2 was M = 6.83 (SD = 6.84; t_(138)_ = 1.31, *p* = 0.192). There was also no significant difference between responders and non-responders of T2 in the recorded GAD symptoms measured at T1 (responder: M = 5.01, SD = 4.64; non-responder T2: M = 4.28, SD = 4.92; t_(140)_ = 0.90, *p* = 0.368). In contrast, PTSD symptoms of those who participated in both measurement times were significantly higher than of non-responders of T2 (responder: M = 15.56, SD = 11.59; non-responder T2: M = 9.47, SD = 10.92; t_(94)_ = 2.63, *p* = 0.010).

### Sample characteristics (sociodemographic and migration-specific variables)

The study sample included 108 Syrian refugees with residence permission, 71 (65.7%) were recognized as persecuted refugees, 30 (27.8%) received subsidiary protection and 7 (6.5%) came to Germany due to the law of family reunion. Thirty-four of the participants were female (31.5%) and 74 male (68.5%). The mean age of the participants at T1 was 35.67 years (SD = 10.93, range: 19–63) and 36.92 years (SD = 10.81, range: 20–64) at T2. At both measurement points, 75 participants (69.4%) were parents, and had a mean of 3.43 children (SD = 1.49; range: 1–11).

Regarding the migration-related variables of our study sample their mean duration of future residence permission in Germany was 15.39 months at T1 (SD = 9.14, range: -9–32) and 9.76 months at T2 (SD = 10.51, range: -2–35). Further sociodemographic and migration-specific variables are shown in Table 1 and gender-specific data for T1 is given in Georgiadou et al. (2018) [32].

**Table 1:** Sociodemographic and migration-specific variables of the study sample at both measurement times (*N* = 108)

***Sociodemographic and migration-specific variables***	**T1**		**T2**	
	**n**	**%**^**ab**^	**n**	**%**^**ab**^
**Age group**				
18–24	18	16.7	16	14.8
25–44	69	63.9	69	63.9
≥ 45	21	19.4	23	21.3
**Marital status**				
Single	29	26.9	29	26.9
Married	74	68.5	74	68.5
In a relationship	1	0.9	1	0.9
Divorced/Widowed	4	3.7	4	3.7
**Accommodation**				
Collective accommodation center	14	13.0	8	7.5
Own apartment (alone or with family)	77	71.3	86	80.4
Shared flat	17	15.7	13	12.2
**Current activities**^**c**^				
German classes	76	70.4	43	39.8
Integration classes	24	22.2	24	22.2
School/Studies	3	2.8	18	16.9
Full-time/part-time job	4	3.7	12	11.1
Marginal employment	3	2.8	8	7.4
Other	15	13.9	18	16.7
**Religion**				
Muslim	100	92.6	100	92.6
Christian	4	3.7	4	3.7
None/Other	4	3.7	4	3.7
	**M (SD)**	**Range**	**M (SD)**	**Range**
**Education in years** (*n* = 107)	9.97 (4.63)	0–20	10.76 (5.53)	0–22
**Duration of stay in Germany**^**d**^ (*n* = 108)	22.81 (6.90)	2–50	42.02 (6.35)	18–67
**Duration of residence permission**^**d**^ (*n* = 98)	31.15 (11.44)	5–75	32.68 (8.43)	8–59
**Future validity of permit**^**d**^ (*n* = 98)	15.39 (9.14)	-9-32^e^	9.76 (10.51)	-2-35^e^

^a^ Valid values; ^b^Add-ups may not be equal to total due to rounding; ^c^ Multiple answers possible; ^d^ In months; ^e^ Negative values for current extension requests

### Life satisfaction and perceived discrimination in Germany

On a visual scale from “not satisfied at all” (0) to “very satisfied” (10), the satisfaction with living in Germany was relatively high indicated as on average 7.56 (SD = 2.41; range: 0–10) at T1 and 7.79 (SD = 2.08; range: 0–10) at T2, with no significant difference (t_(107)_ = −.524, *p* = 0.602).

The participating Syrian refugees on average rarely perceived discrimination but significantly more at T2 than at T1 (T1: M = 0.52, SD = 0.79; T2: M = 0.83, SD = 0.93; t_(107)_ = − 3.652, *p* < 0.001). Of the four areas surveyed - “neighborhood”, “shops”, “authorities” and “professional life” - only the “neighborhood” category showed significant changes (T1: M = 0.46, SD = 0.79; T2: M = 0.83, SD = 1,01; t_(107)_ = − 3.998, *p* < 0.001).

### Change in the prevalence of depression, generalized anxiety symptoms, experienced traumatic events, and PTSD

The total score for depression at T1 was M = 7.41 (SD = 6.38, range: 0–25) and M = 7.01 (SD = 5.49, range: 0–22) at T2 with no significant difference (t_(108)_ = 0.75, *p* = 0.453). In total, 29 participants (26.9%) at T1 and 33 participants (30.6%) at T2 reported clinically relevant depressive symptoms. For generalized anxiety disorder it was 18 participants (16.7%) at T1 and 17 (15.7%) at T2 with a clinically relevant anxiety score. The total score at T1 was on average M = 5.07 (SD = 4.88, range: 0–18) and M = 4.83 (SD = 4.76, range: 0–21) at T2. Again, there was no significant difference between baseline and follow-up (t_(108)_ = 0.55, *p* = 0.581).

At T1, 78 participants (72.2%) and at T2 84 participants (77.8%) reported having experienced and/or witnessed at least one traumatic event. This corresponds to an significant increase (*χ*^*2*^_(1)_ = 10.71, *p* = 0.001). On average, participants at T1 had experienced and/or witnessed M = 2.38 (SD = 2.52) traumatic events with a significant increase to T2 (M = 2.93, SD = 2.59; t_(108)_ = − 2.96, *p* = 0.004). For the participants that had experienced at least one traumatic event at T1 there was no significant difference for the severity of PTSD symptoms from T1 to T2 (T1: M = 15.56, SD = 12.85, range: 0–48; T2: M = 13.35, SD = 12.27, range: 0–51; t_(78)_ = 0.13, *p* = 0.209). Indication of the presence of PTSD at T1 were found in 15 participants (13.9%) and 14 participants (13.0%) at T2. Detailed data for severity of depression, generalized anxiety disorder and PTSD are reported in Table 2.

**Table 2:** Depression, generalized anxiety disorder, and PTSD and their distribution at both measurement times (*N* = 108)

	T1		T2		Comparison	
	n	%^**f**^	n	%^**f**^	***z***^***g***^	***p***
**Depression**^**a**^						
Clinically not relevant (< 10)	79	73.1	75	69.4	−0.282	0.778
Moderate (10–14)	11	10.2	21	19.4		
Moderately severe or severe (≥ 15)	18	16.7	12	11.1		
**Generalized anxiety disorder**^**b**^						
Clinically not relevant (< 10)	90	83.4	91	84.3	−0.136	0.892
Moderate (10–14)	10	9.3	12	11.1		
Severe (≥ 15)	8	7.4	5	4.6		
**PTSD**^**c, d**^						
Clinically not relevant	41	38.0	41	38.0	−0.346	0.730
Clinically marginally relevant	22	20.4	23	21.3		
Clinically relevant	15	13.9	14	13.0		
**Number of mental disorders**^**e**^						
Without mental disorder	74	68.5	68	63.0	−0.273	0.787^d^
One mental disorder	15	13.9	22	20.4		
Two mental disorders	10	9.3	12	11.1		
Three mental disorders	9	8.3	6	5.6		
**Single diagnostic category**						
Only depression^a^	11	10.2	7	6.5		
Only GAD^b^	0	0.0	1	0.9		
Only PTSD^c^	3	2.8	4	3.7		
**Two diagnostic categories**						
Depression^a^ and GAD^b^	8	7.4	4	3.7		
Depression^a^ and PTSD^c^	1	0.9	2	1.9		
GAD^b^ and PTSD^c^	1	0.9	0	0.0		
**Three diagnostic categories**						
Depression^a^, GAD^b^ and PTSD^c^	9	8.3	6	5.6		

^a^ PHQ-9, Patient Health Questionnaire – Depression Module; ^b^ GAD-7, Generalized Anxiety Disorder Scale; ^c^ ETI, Essen Trauma Inventory: sum score calculated on the basis of the three subscales (intrusion, hyperarousal and avoidance) that are relevant for the cut-off for a PTSD diagnosis; ^d^ PTSD was only calculated for those participants that had already experienced at least one traumatic event at T1 (*n* = 78); ^e^ Depression (PHQ-9 total score ≥ 10), generalized anxiety disorder (GAD-7 total score ≥ 10), PTSD (ETI: sum score calculated on the basis of the three subscales (intrusion, hyperarousal and avoidance) that are relevant for the cut-off for a PTSD diagnosis); ^f^ Add-ups may not be equal to total due to rounding; ^g^ Wilcoxon signed-rank test

Regarding the distribution of the number of diagnoses from T1 to T2 no significant differences showed up (*z* = − 0.27, *p* = .787). The change in the distribution of the number of mental disorders as well as the frequencies of morbidities and comorbidities of mental disorders in the study sample are also presented in Table 2.

### Previous treatment for mental disorders

At T1, seven participants (6.5%) stated having already been treated for psychological complaints. About 1.5 years later at T2, it was only one more participant (*n* = 8, 7.4%). The self-mentioned main reasons for the treatment were depression, insomnia, poor concentration, rumination and restlessness.

### Change in predictors of depression, generalized anxiety and PTSD symptoms

Multiple regression analyses were used to examine the potentially varying influence of several sociodemographic and migration-specific variables as well as traumatic experiences on the severity of depression, generalized anxiety and PTSD symptoms with increasing length of stay in Germany (Table 3). For depression symptoms a significant regression equation was found at T1 (F (9,80) = 6.018, *p* < .001), with an explanation of variance of 33.7% and at T2 (F (9,95) = 5.745, *p* < .001), with 29.1%. At both times of measurement, perceived discrimination (T1: β = 0.235, *p* = 0.027; T2: β = 0.271, *p* = 0.008) and the number of traumatic experiences (T1: β = 0.562, *p* < 0.001; T2: β = 0.362; *p* < 0.001) were significant predictors of depressive symptoms. At T1, fewer years of education (β = − 0.263, *p* = 0.007) and at T2 female gender (β = − 0.246, *p* = 0.014) were also significantly related to stronger symptoms of depression. When increased symptoms of generalized anxiety were predicted, it was found that at T1 and T2 female gender, higher perceived discrimination and higher numbers of traumatic events were significant predictors. The explained variance was 34.6% (F (9,80) = 6.239, *p* < .001) for T1 and 27.6% (F (9,95) = 5.405, *p* < .001) for T2. At T1 female gender significantly predicted stronger symptoms of PTSD (β = − 0.252, *p* = 0.017), as did a higher amount of traumatic events (β = 0.478, *p* < 0.001). The results of the regression indicated an explained variance of 28.2% (F (9,80) = 4.885, *p* < .001). At T2, more severe symptoms of PTSD were significantly associated with shorter validity of the residence permission (β = − 0.184, *p* = 0.020) and, same as at T1, higher numbers of traumatic experiences (β = 0.557, *p* < 0.001). The regression equation of this model was significant (F (9,95) = 8.199, *p* < .001), with an explanation of variance of 38.4%.

**Table 3:** Multiple linear regression analyses predicting symptoms of depression, generalized anxiety and PTSD at T1 and T2 (*N* = 108)

Predictors of depressive symptoms^***a***^	B^***d***^	95% CI^***e***^	SE^***f***^	β	***p***
T1*					
Years of education	−0.360	−0.619 to −0.101	0.130	−0.262	0.007
Perceived discrimination	1.885	0.219 to 3.552	0.837	0.235	0.027
Number of traumatic events	1.421	0.934 to 1.909	0.245	0.562	< 0.001
T2*					
Gender^*g*^	−2.905	−5.474 to −0.904	1.157	−0.246	0.014
Perceived discrimination	1.599	0.383 to 2.769	0.595	0.271	0.008
Number of traumatic events	0.769	0.381 to 1.157	0.194	0.362	< 0.001
**Predictors of GAD symptoms**^***b***^					
T1*					
Gender^*g*^	−4.549	−6.663 to −2.434	1.062	−0.421	< 0.001
Perceived discrimination	1.674	0.359 to 2.988	0.660	0.263	0.013
Number of traumatic events	0.817	0.432 to 1.202	0.193	0.406	< 0.001
T2*					
Gender^*g*^	−3.463	−5.563 to −1.659	1.001	−0.342	0.001
Perceived discrimination	1.689	0.700 to 2.738	0.515	0.335	0.001
Number of traumatic events	0.421	0.096 to 0.758	0.167	0.232	0.014
**Predictors of PTSD symptoms**^***c***^					
T1*					
Gender^*g*^	−7.131	−12.930 to −1.332	2.914	−0.252	0.017
Number of traumatic events	2.515	1.460 to 3.570	0.530	0.478	< 0.001
T2*					
Future validity of permit^*h*^	−0.211	−0.388 to −0.035	0.089	−0.184	0.020
Number of traumatic events	2.583	1.800 to 3.365	0.394	0.557	< 0.001

* The following variables were included in each of the calculated multiple regressions at T1 and T2: gender, age, years of education, accommodation, work/school/studies, duration of stay in Germany, future validity of permit, perceived discrimination and number of traumatic events. In this table only significant predictors are shown

^a^ PHQ-9, Patient Health Questionnaire - Depression Module, sum score; ^b^ GAD-7, Generalized Anxiety Disorder Scale, sum score; ^c^ ETI, Essen Trauma Inventory, sum score calculated on the basis of the three subscales (intrusion, hyperarousal and avoidance) that are relevant for the cut-off for a PTSD diagnosis; ^d^ B = regression coefficient; ^e^ CI = confidence interval; ^f^ SE = standard error; ^g^ 0 = female, 1 = male; ^h^ In months

## Discussion

To the best of our knowledge, this is the first register-based study to have followed up the mental health, living conditions and potential stress factors of a sizeable group of Syrian refugees with residence permission in Germany. The 1.5 year follow-up revealed that mental distress does not change significantly over time, and overall remains quite high in comparison to the general population in Germany [42–44]. However, compared to other studies with Syrian refugees, the observed prevalence rates of depression, PTSD and anxiety disorder were much lower [11, 16]. Experienced traumatic events as well as post-migration factors, such as discrimination or further validity of residence permission were both identified as relevant risk factors for mental disorders.

Concerning demographic properties of our sample, most of the 108 included Syrian refugees were male (68.5%) and middle-aged (M = 35.3 at T1). As in the period from 2014 to 2017, 64.8% of requests for asylum from Syrian refugees were made by men, this is comparable to our sample [45–47]. Relative to the age structure of asylum seekers over the age of 18 in Germany in this period of time, the majority of applications were made by asylum seekers aged between 18 and 44 (70.6%) [45–47]. In our study, 80.6% of participants were aged between 18 and 44. With regard to the age structure, our sample is also comparable to that of the total population of applicants in Germany. The average duration of previous residence in Germany was 1.9 years at T1 and 3.5 years at T2. In the meantime, from T1 to T2, 9% of the participants, resident in collective accommodation centers or shared flats, have moved into their own apartments (alone or with family).

The largest change concerning sociodemographic variables has been found among the refugees’ current activities. While only 9.3% of the participants went to school, university or were employed at T1, the rate was 35.4% for T2. These changes indicate an integration process of our sample. But, it must be taken into consideration, that 40% of the employed participants are only marginally employed and therefore cannot make a living from their work.

As shown in previous surveys, the presented study confirms the high satisfaction of refugees with living in Germany (on average between 7.6 and 7.8 on a scale of 1–10) [21]. In a representative survey carried out in Germany, the 4.500 asked refugees reported values similar to those of the general population in the subjective assessment of their life satisfaction. Comparable to the examined values in our study, the mean was about 7 on a scale of 1–10 [48]. In addition, the low values of perceived discrimination also correspond to earlier study results on refugees in Germany [21]. Among the 4.500 refugees in the German study presented, a minority of 10% reported frequent experiences of discrimination and another 36% rarely [48]. Even though only rare discrimination experiences were reported at both measurement times, the total discrimination as well as the perceived discrimination in the neighborhood at T2 were significantly higher than at T1. This may possibly be explained by the change in living conditions. In comparison to T1, at T2 considerably more Syrian refugees lived in their own apartments, which may have led to more contact with locals and discriminatory experiences in the neighborhood.

In line with previous studies on Syrian refugees, high rates of traumatizing experiences are reported [3, 6, 20]. With a longer duration of stay in Germany, a significant increase of having experienced and/or witnessed traumatic events as well as in the number of traumatic events mentioned was found. Different reasons for this increase could be possible. Firstly, this result indicates traumatic experiences Syrian refugees went through whilst staying in Germany, or could point to evolutions in remembering certain traumatic events, or might be an indication of memory inconsistency, possibly caused by mental health problems [49].

In our sample, 26.9% fulfilled the criteria for a clinically relevant depression diagnosis, 16.7% for a generalized anxiety disorder and 13.9% for PTSD at T1. Approximately 1.5 years later (at T2), these prevalence rates hardly changed and were 30.6% for depression, 15.7% for generalized anxiety and 13.0% for PTSD. None of these diagnoses revealed a significant increase or decrease after this time. A comparison of the number of psychological complaints of T1 and T2 neither showed significant changes. While at T1 31.5% met the criteria for at least one diagnosis, at T2 it was 37.0%. Regardless of baseline or follow-up, these findings are in contrast to previous study results from different countries and different points of the resettlement process that have shown considerably higher prevalence rates of mental disorders among Syrian refugees [16–21, 23].

A large part of this discrepancy could possibly be explained by clinical and methodological factors, in terms of differences in sampling and assessment methods [30]. For example, Acarturk et al. [20] found a particularly high probable prevalence rate for PTSD of 83.5% among the examined Syrian refugees located in a refugee camp in Turkey. That result could be attributed to the short duration of the sample’s stay in Turkey (about one year) and the location of the camp at the border of Syria. In contrast, the lower prevalence rates of our study sample may be related to the safe environment with good living conditions in Germany, the longer length of stay and the possession of residence permission. Many studies with higher prevalence rates have focused on asylum seekers instead of refugees with residence permission [4, 6, 31, 50]. Besides, the countries of resettlement, including their associated living conditions may have an impact on mental disorders. Compared to other countries in which studies on the health status of Syrian refugees have already been carried out, Germany is characterized by a low unemployment rate (about 5.1%), high living standards and a health system based on sharing in which 97% of the population is covered by health insurance [51–53]. Overall, there are a lot of pre- and post-migration factors as well as methodological differences that play a role in the frequency of prevalence rates of psychological distress between studies.

Regarding the persistent mental health problems of our study sample over time, our results correspond to previous research on the influence of length of stay in receiving countries on psychological stress among refugees. Several studies have confirmed the persistence of mental problems even after more than three or five years in the host country [30, 54]. On the one hand, a path analysis performed to investigate post-migration psychological symptoms among refugees in Germany found on average similar PTSD and depressive symptoms over the course of one year [27]. On the other hand, a previous study by our group on Arabic-speaking asylum seekers suggested participants with mental distress have shorter periods of stay in Germany compared to the asylum seekers without mental distress. In this study, mental health of some asylum seekers was better with a longer duration of stay in Germany. However, the difference in length of stay between asylum seekers with and without psychological stress was only around four months [3]. Contrary findings are presented in a study of 225 newly resettled Iraqi refugees in Australia and 225 Iraqis with a period of resettlement of approximately five years. A significant difference between groups in symptoms of anxiety and depression was found, indicating that study participants with longer periods of resettlement were experiencing higher levels of psychological distress than recent arrivals [55]. This might be a proof of the strong influence of post-migratory variables on mental distress. In summary, follow-up studies with the same participants over time showed no significant changes with an increased length of stay, whereas some comparative studies between different samples showed significant differences. An attribution of these different results to the study design may be possible. With regard to our study sample, ongoing post-migratory stress, such as discrimination and poor integration or the lack of treatment may be reasons for the consistent prevalence rates of mental disorders at T1 and T2.

Asked about previous treatments due to psychological stress, only 6.5% at T1 and 7.4% at T2 reported having already received psychological help. Given the considerably high prevalence rates of mental disorders found in this study (compared to the German general population), access barriers to mental health services, feelings of shame or lack of information about potential treatment offers or psychological disorders per se may be reasons for the small number of our Syrian refugee sample having received psychotherapeutic treatment so far.

In the next step of our analyses, we investigated the impact of several risk factors from sociodemographic variables, traumatic experiences and post-migration variables on the severity of depressive-, generalized anxiety-, and PTSD symptoms. Regarding the predictors for severity of depressive symptoms at baseline (T1) as well as at follow-up (T2) significant relations were found with higher perceived discrimination and a higher number of traumatic events. In deviation from T2, in T1 less years of education had a significant influence. In T2 women reported stronger depression symptoms than men. Female gender as well as higher perceived discrimination and a higher amount of experienced and/or witnessed traumatic events were identified as significant predictors for higher severity of generalized anxiety. Although female sex was only a significant risk factor for more severe PTSD symptoms at T1, a tendency in this direction was also discernible at T2. In addition, shorter future validity of residence permission at T2 and a higher number of traumatic events at both measurement times were significantly associated with reported PTSD symptoms. The shorter future validity as predictor for PTSD symptoms at T2 may point to the uncertainty about their future stay in the host country and the corresponding fear about returning to their problematic home country [32]. These results strongly indicate that both traumatic experiences as well as daily stressors such as discrimination have a large impact on the mental health development of Syrian refugees in Germany, as already suggested in other studies [9, 18, 20, 24, 31]. Both factors need to be considered as important aspects impacting on refugees’ mental health. We found little evidence for possible changes in the impact of traumatic experiences and post-migration aspects over time. We hypothesize traumatic experiences would have a larger impact on the shorter and daily stressors in the long term; hereby a longer follow-up study is needed to investigate this hypothesis further.

### Strengths, limitations and implications for further research

This is the first register-based follow-up study of mental disorders among Syrian refugees with residence permission in Germany. Our design clearly stands out in comparison to previous research on refugees’ mental health. Other strengths can be found in the comparatively high response rate from baseline to follow-up despite the costly recruiting method with prearranged appointments. The good comparability to the basic population of Syrian refugees in Germany as well as the inclusion of selected pre- and post-migration variables in the analyses should also be emphasized.

Despite these strengths there are also some limitations to our study. Non-responders at T2 were significantly younger and had lower severity of PTSD symptoms which could represent a selection bias among the study sample. Our study is based on self-rating scales and therefore does not fulfill the gold standard criteria of clinical diagnosis. Due to these self-report assessments, the presented results could be biased by social desirability. In addition, not all of the possible psychiatric comorbidities, such as drug abuse could be detected. In literature, we find remarks on comorbid occurrence of PTSD and substance use disorder [56]. Besides, our study includes only a 1-time follow-up at a 1.5 year interval. Even if the presented results indicate a high persistence of mental disorders among Syrian refugees with residence permission, longer follow-up studies should verify these findings, screen for a broader spectrum of mental problems, and also for more protective and risk factors. Especially post-migration variables should become a stronger focus of research. In addition, barriers to accessing mental health services as well as mental disease concepts should be assessed. It is important to determine the prevalence of common stress factors, and provide needed interventions, as these conditions, if untreated, can be detrimental to mental health. We also suggest implementing interventional follow-up studies with trauma therapeutic concepts to examine the potential effect on Syrian refugees’ mental disorders. For the therapeutic treatment of Syrian refugees, we recommend a culture sensitive, individualized approach, which takes into account traumatic as well as post-migratory aspects. Due to the low uptake of therapeutic services, training courses would also be useful to make the refugees aware of mental distress and its risk factors. In addition, the integration process of especially female refugees should be therapeutically accompanied and refugee aid-workers should be trained on the specific burdens of refugees [57].

## Conclusions

There is strong empirical evidence that the prevalence rates of mental distress among refugees are significantly higher compared to the overall population, even if the prevalence rates of psychological disorders among this survey cohort were not as high as in previous studies with the Syrian refugee population [20]. However, it has not yet become clear if and how these prevalence rates change with an increasing length of stay in the host countries. The results of our study indicate that the psychological burden on Syrian refugees with residence permission in Germany remains consistently high over time, despite partly improved living conditions. The most vulnerable group among the Syrian refugees is characterized by female gender, a high number of experienced traumatic events, and frequently perceived discrimination and call for specific attention. The persistently high values of psychological stress confirm the importance of therapeutic interventions for Syrian refugees.

## Data Availability

The dataset analyzed during the current study is available from the corresponding author on reasonable request.

## Abbreviations

T1: First measurement point
T2: Second measurement point
PTSD: Post-traumatic stress disorder
GAD: Generalized anxiety disorder
B: Regression coefficient
CI: Confidence interval
SE: Standard error
